# Relationships between immune gene expression and circulating cytokine levels in wild house mice

**DOI:** 10.1002/ece3.6976

**Published:** 2020-11-09

**Authors:** Stuart Young, Jonathan Fenn, Elena Arriero, Ann Lowe, Benoit Poulin, Andrew D.C. MacColl, Janette E. Bradley

**Affiliations:** ^1^ School of Life Sciences University of Nottingham Nottingham UK; ^2^ North of England Zoological Society Chester UK; ^3^ Department of Biodiversity, Ecology and Evolution University Complutense of Madrid Madrid Spain; ^4^ Leicester Biomedical Research Centre University Hospitals of Leicester NHS Trust General Hospital Leicester UK

**Keywords:** cytokines, ecoimmunology, gene expression, immunity, immunology, qPCR, RT‐qPCR, wild immunology

## Abstract

1. Quantitative PCR (qPCR) has been commonly used to measure gene expression in a number of research contexts, but the measured RNA concentrations do not always represent the concentrations of active proteins which they encode. This can be due to transcriptional regulation or post‐translational modifications, or localization of immune environments, as can occur during infection. However, in studies using free‐living non‐model species, such as in ecoimmunological research, qPCR may be the only available option to measure a parameter of interest, and so understanding the quantitative link between gene expression and associated effector protein levels is vital.
2. Here, we use qPCR to measure concentrations of RNA from mesenteric lymph node (MLN) and spleen tissue, and multiplex ELISA of blood serum to measure circulating cytokine concentrations in a wild population of a model species, *Mus musculus domesticus*.
3. Few significant correlations were found between gene expression levels and circulating cytokines of the same immune genes or proteins, or related functional groups. Where significant correlations were observed, these were most frequently within the measured tissue (i.e., the expression levels of genes measured from spleen tissue were more likely to correlate with each other rather than with genes measured from MLN tissue, or with cytokine concentrations measured from blood).
4. Potential reasons for discrepancies between measures including differences in decay rates and transcriptional regulation networks are discussed. We highlight the relative usefulness of different measures under different research questions and consider what might be inferred from immune assays.

## Graphical abstract

In wild house mice, there is little correlation between immune gene expression and final proteins. This is important to consider in future ecoimmunology research.

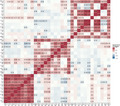

## 1 INTRODUCTION

Since the field of ecoimmunology emerged over two decades ago (Sheldon & Verhulst, 1996), measuring the immune function of wild animals has remained challenging (Pedersen & Babayan, 2011). To date, the vast majority of ecoimmunological studies have used non‐model species (Jackson, 2015) and so have been limited in their scope or by the availability of appropriate assays or reagents (Fassbinder‐Orth, 2014; Zimmerman et al., 2014). Because of this, many early studies relied on less specific immunological assays, such as leukocyte counts, hemagglutination, responses after stimulation with phytohemagglutinin, and bactericidal assays (Demas et al., 2011; Pedersen & Babayan, 2011; Zimmerman et al., 2014).

As modern sequencing technologies have become more accessible and affordable, it has become possible to measure immune responses in non‐model organisms using more specific assays (Jackson, 2015). For example, reverse transcription quantitative real‐time PCR (RT‐qPCR, often abbreviated to qPCR) can be used to measure the expression level of immune genes, such as cytokines, via mRNA concentrations (Adams, 2020). This gene expression‐based approach has been widely used to investigate immune function in wild populations of a range of taxa (Fassbinder‐Orth, 2014).

Gene expression is regularly used as a proxy for measuring functional protein products (Fassbinder‐Orth, 2014), and it is often assumed that the levels of mRNA expression will correlate with the levels of the corresponding proteins (Fassbinder‐Orth, 2014; Maier et al., 2009; Vogel & Marcotte, 2012). Indeed, many researchers have focused solely on qPCR and measuring levels of gene expression (Koussounadis et al., 2015). However, the correlation between mRNA concentration and corresponding protein concentration is often dependent on cell type and state (Silva & Vogel, 2016). Indeed, in bacteria and eukaryotes only around 40% of variation in cellular protein levels can be predicted by mRNA concentrations (de Sousa Abreu et al., 2009; Vogel & Marcotte, 2012). Therefore, mRNA concentrations may not always accurately represent the expression levels of the bioactive protein (Jackson, 2015; Pradet‐Balade et al., 2001) Further, the immune response may be localized and tissue specific, for example, in response to localized infections or tissue damage (Hu & Pasare, 2013).

Although measures of immune gene expression in vertebrates may indicate an induced immune response in the host (Jackson, 2015), it should not necessarily be assumed that levels of gene expression completely align with their corresponding effector proteins. In addition to localization of responses, there may be levels of regulation occurring between genes, or delays between transcription and translation and protein modification (Payne, 2015; Pradet‐Balade et al., 2001). Indeed, studies have found that the extent to which mRNA expression correlates with the final gene products can vary with transcriptional and post‐translational processes, and that the rates of decay between the two can significantly differ (Munsky et al., 2012; Schwanhäusser et al., 2011). Therefore, making robust measures of molecular phenotype requires the measurement of active proteins using antibody reagents against key immunological biomolecules (Jackson, 2015). This is particularly important when dealing with natural populations in which variation is intrinsically higher (Abolins et al., 2011, 2017).

Protein levels can only reliably be analyzed using sophisticated assays that are typically only available for model species, such as house mice (*Mus musculus*). For these species, commercial off‐the‐shelf reagents are readily available. Due to structural variation in immune molecules such as cytokines, however, the transferability and cross‐reactivity of these assays to non‐model species are extremely limited (Jackson, 2015). Developing specific antibodies is typically expensive and time‐consuming (Bradley & Jackson, 2008; Friberg et al., 2010; Jackson, 2015; Oko et al., 2006; Pedersen & Babayan, 2011). Therefore, it is informative to understand the precise relationships between measured gene expression and the effector proteins that will actually interact at the immune interface with a pathogen or parasite, particularly in wild populations.

Despite the inherent difficulties associated with applying sophisticated molecular techniques to wild animals, a number of studies have investigated the immune function of wild populations of model species, primarily house mice. These have generally employed specific measures of immune function, such as antibody concentrations and avidity (Abolins et al., 2011; Lochmiller et al., 1991), or circulating cytokine concentrations (Abolins et al., 2017).

Here, using the extensively studied natural population of *M. m. domesticus* on the Isle of May (for examples see Berry et al., 1990; Goertz et al., 2019; Taylor et al., 2019; Triggs, 1991), we used qPCR to measure the expression levels of key cytokine genes representing four arms of the adaptive immune response (Th1, Th2, Treg, and Th17), as well as the innate immune response. These were measured from two key immunological tissues; the spleen and mesenteric lymph nodes (MLN). In addition, we measured the circulating levels of the corresponding secreted cytokines from serum using multiplex bead assay. Further, the relative mRNA expression of several important immunity‐related transcription factors was measured using qPCR from spleen and MLN tissue.

We aimed to assess the relationship between the expression of these key cytokine genes and the corresponding circulating protein levels in our population of *M. m. domesticus*. Further, by using both MLN and spleen tissue, we aim to explore the relationship between immune gene expression from two distinct but immunologically important tissue types.

Due to post‐translational processes and protein degradation, we hypothesized that there would not be widespread correlation between gene expression and circulating cytokine levels. The expression level of genes from MLN tissue, for example, are unlikely to relate to the expression level of genes from spleen tissue or concentrations of circulating cytokines as these represent a localized, gastrointestinal response. Further, we hypothesized that there may be antagonistic relationships between some response types. For example, the Th1‐type response would not be expected to correlate with the Th2‐type response due to their antagonistic relationship (Kaiko et al., 2008).

## 2 Methods

### 2.1 Sample collection

Mice were live‐trapped from a wild population on the Isle of May (56°11′11.6″N, 2°33′24.1″W), located approximately 8 km off the southeast coast of Scotland in the Firth of Forth. Trapping was conducted across 8 sessions from August 2014 to October 2016, with each session including 4–7 trapping days. Trapping transects were placed in up to 11 locations across the island (Taylor et al., 2019). Longworth traps (Longworth Scientific Instrument Co.) were primarily used, along with small numbers of Ugglan (Granhab) and homemade “Jordan” traps (Perrow & Jowitt, 1995). Mice were euthanized in a CO_2_ chamber with a rising gradient of CO_2_. The eye and foot reflexes were tested, and death was confirmed through exsanguination by cardiac puncture. Immediately following the confirmation of death, a sample of blood was removed to a 1.5 ml microcentrifuge tube. This was stored at room temperature for ~60 min and then stored at 4°C for a further ~60 min. The resulting clot was carefully removed, and the remaining serum was spun at 3,000 g for 10 min. The serum was removed and snap frozen using liquid nitrogen, before being stored at −80°C until its use in multiplex bead assays to measure cytokine levels.

The spleen and MLN were removed and placed in RNAlater solution (Life Technologies). Following the manufacturer's recommendations, tissues were sectioned into smaller pieces (≤ 5 mm) prior to immersion in the solution. Samples were kept at 4°C for 24 hr, then the supernatant was removed, and samples were stored at −80°C until RNA extraction.

### 2.2 RNA extraction & cDNA synthesis

RNA was extracted from up to 30 mg spleen and MLN tissue stored in RNAlater using the NucleoSpin RNA kit (Machery‐Nagel), following the manufacturers’ protocol. A DNase treatment step is included in this protocol, and RNA was eluted in 60 μL of nuclease‐free water. The purity and concentration of RNA were assessed on a NanoDrop 1,000 spectrophotometer (Thermo Scientific), with a desired 260/280 absorbance ration of >1.80 (Robertson et al., 2016). To check for contamination, and to assess the purity of RNA, 6 μL of each sample was mixed with 4 μL of Orange G gel loading dye (Sigma Aldrich). This was incubated at 65°C for 10 min, followed by visualization on a 2% agarose gel stained with ethidium bromide run at 90V for 30 min.

Synthesis of cDNA was performed on up to 2 µg of total RNA using the nanoScript2 Reverse Transcription kit (Primerdesign), using a combination of oligo‐dT and random nonamer primers (0.5 µl of each). Nuclease‐free water was used to make a total reaction volume of 10 µl. This was incubated at 65°C for five minutes, before being cooled on ice. To this, 10 µl of extension‐mix (5.0 µl of nanoScript2 4X Buffer, 1.0 µl of 10 mM dNTP mix, 1.0 µl of nanoScript2 enzyme and 3.0 µl of nuclease‐free water) was added. This was incubated at 25°C for five minutes then 42°C for 20 min, before being heat inactivated at 75°C for ten minutes. No‐enzyme controls were used to monitor samples for contamination by genomic DNA. All cDNA samples were diluted 1:10 with nuclease‐free water and then stored at −20°C for future analysis by qPCR.

### 2.3 qPCR conditions

qPCR was carried out to measure normalized mRNA expression in spleen and MLN tissue of a suite of genes reflecting different functional arms of the immune system. mRNA levels of seven cytokine genes of interest were measured from both spleen and MLN tissue including Tumor necrosis factor‐α (TNF‐α), Interferon‐γ (IFN‐γ), Interleukin (IL)‐1β, IL‐6, IL‐10, IL‐12β, IL‐13, and IL‐17. Expression levels of IL‐5 were also measured from spleen tissue, but not MLN (Table 1). Further, the relative mRNA expression of several important immunity‐related transcription factors was measured using qPCR, from both spleen and MLN tissue. This included expression levels of GATA binding protein 3 (Gata3), Interferon regulatory factor 5 (Irf5) and T‐box 21/Tbet (Tbx21) (Table 1). All primers used were designed and validated by Primerdesign (Southampton). The genes analyzed, along with their corresponding GenBank accession numbers and primer sequence information, are summarized in Table 1. Each qPCR reaction contained 0.5 µl of the relevant primer mix (Table 5.1), 2.5 µl of template cDNA, 2.0 µl of nuclease‐free H_2_O and 5 µl PrecisionFAST qPCR Mastermix with LOW ROX (Primerdesign), using SYBR Green chemistry, to make a total reaction volume of 10 µl. All samples were run in duplicate, with each plate also containing negative controls and a pooled reference sample.

**Table 1:** Primer details for qPCR assays of a suite of cytokines and immune‐related transcription factors in wild house mice (*Mus musculus domesticus*) from the Isle of May

Gene	Accession number	Primer sequence	Amplicon size (bp)	Single exon	Tm (^o^C)	Function	Molecule type	Tissue
IL−1β *Il1b*	NM_008361	*L* 5’‐CAACCAACAAGTGATATTCTCCAT	127	Yes	73.3	Innate inflammation (Jackson et al., 2011)	Cytokine	MLN; Spleen
		*R* 5’‐ GGGTGTGCCGTCTTTCATTA						
Irf5 *Irf5*	NM_001252382	*L* 5’‐CTTCCAGCCAGCCCCCTA	84	ND	75.3	Induces innate immune response (Ban et al., 2018)	Transcription factor	MLN; Spleen
		*R* 5’‐TGATCTCTAGGTCCGTCAAAGG						
IFN‐γ *Ifng*	NM_008337	*L* 5’‐TGATTACTACCTTCTTCAGCAACAG	128	No	74.5	Upregulation of Th1 response (Turner et al., 2011)	Cytokine	MLN; Spleen
		*R* 5’‐CTGGTGGACCACTCGGATG						
IL−12β *Il12b*	NM_008352	*L* 5’‐CACGGCAGCAGAATAAATATGAG	106	No	72.9	Pro‐inflammatory Th1 (Turner et al., 2011)	Cytokine	MLN; Spleen
		*R* 5’‐GAGTTCTTCAAAGGCTTCATCTG						
Tbx21 *Tbx21*	NM_019507	*L* 5’‐AACCAGCACCAGACAGAGAT	120	ND	76.5	Th1 inflammation (Mullen et al., 2001)	Transcription factor	MLN; Spleen
		*R* 5’‐GACCACATCCACAAACATCCT						
TNF‐α *Tnf*	NM_013693	*L* 5’‐AGCCAGGAGGGAGAACAGA	96	Yes	73.8	Th1 inflammation (Britt et al., 2019)	Cytokine	MLN; Spleen
		*R* 5’‐CAGTGAGTGAAAGGGACAGAAC						
IL−5 *Il5*	NM_010558	*L* 5’‐ATGGACGCAGGAGGATCAC	128	No	74.5	Upregulation of Th2 response (Turner et al., 2011)	Cytokine	Spleen
		*R* 5’‐TTGAAGTTAGATAGGAGCAGGAAG						
IL−13 *Il13*	NM_008355	*L* 5’‐GCCAGCCCACAGTTCTACA	98	ND	78.1	Upregulation of Th2 response (McKenzie et al., 1998)	Cytokine	MLN; Spleen
		*R* 5’‐CCACCAAGGCAAGCAAGAG						
Gata3 *Gata3*	NM_008091	*L* 5’‐GAAGACTTTATTGTACCTGGATAGC	118	ND	70.5	Th2 effector responses (Zhu et al., 2006)	Transcription factor	MLN; Spleen
		*R* 5’‐TGGACATCAGACTTAGTGGTTTC						
IL−6 *Il6*	NM_031168	*L* 5’‐TCCATCCAGTTGCCTTCTTG	106	Yes	76.0	Pro‐inflammatory Th17 response (Ambrosi et al., 2012)	Cytokine	MLN; Spleen
		*R* 5’‐GGTCTGTTGGGAGTGGTATC						
IL−17 *Il17a*	NM_010552	*L* 5’‐GTCTGCCCTCCACAATGAAA	75	Yes	68.9	Pro‐inflammatory Th17 response (Ambrosi et al., 2012)	Cytokine	MLN; Spleen
		*R* 5’‐TTAAAGTCCACAGAAAAACAAACAC						
IL−10 *Il10*	NM_010548	*L* 5’‐GGGAAGAGAAACCAGGGAGAT	102	Yes	75.8	Anti‐inflammatory Treg response (Jackson et al., 2011)	Cytokine	MLN; Spleen
		*R* 5’‐GCCACAGTTTTCAGGGATGA						

Note

“Gene” gives the gene name and official gene symbol (in italics). “Accession number” gives the gene on which primers were based. “Amplicon size” indicates the size of the product in base pairs. “Single exon” shows where the primers cover a single exon (“Yes”), span an intron (“No”), or where these data are not determined (“ND”). “Tm” shows the primer melting temperature. “Function” summarizes the immunological role of the target gene. Gene expression was measured from either spleen or mesenteric lymph node (MLN) tissue.

All assays were run using 96‐well optical PCR plates with optical seals (StarLab) in an ABI 7500 FAST real‐time thermocycler (Applied Biosystems). Each reaction included an initial enzyme activation step of 95°C for 20 s, followed by 40 cycles of a further denaturation step of 95°C for 3 s, then an annealing step of 60°C for 30 s. All runs included a post‐PCR melt curve stage consisting of 95°C for 15 s, 60°C for 1 min, 95°C for 15 s and finally 60°C for 15 s.

### 2.4 Quantification of gene expression

The immunological target genes analyzed here (Table 1) were normalized to the endogenous control genes *Sdha* (Accession number: NM_023281.1; Amplicon size: 181bp) and *Ubc* (Accession Number: NM_019639.4; Amplicon size: 178bp). For cDNA from both spleen and MLN tissue, these were selected as the most stable combination of reference genes from a panel of six candidate endogenous genes (also including *Actb*, *Gapdh, Rn18s,* and *Rpl13a*) using the geNorm algorithm (Vandesompele et al., 2002). The stability of expression was analyzed using qbase+ (Biogazelle). In this geNorm assay, 15 randomly selected spleen or MLN cDNA samples were included.

The expression of each target in each sample relative to expression in the reference cDNA was calculated using the 2−^ΔΔ^ *^C^_T_* method (Livak & Schmittgen, 2001; Pfaffl, 2001; Winer et al., 1999), standardized against the geometric mean *C_T_* of the two reference genes for each sample (Vandesompele et al., 2002).

### 2.5 Multiplex ELISA bead assay

To determine serum cytokine levels, diluted serum samples were processed in duplicate using a custom Bio‐Rad Bio‐Plex Pro mouse cytokine multiplex kit (magnetic bead‐based multiplex immunoassay) according to manufacturer's protocol (Bio‐Rad). Included in the custom multiplex panel were antibodies for the detection of the following mouse cytokines: IFN‐γ, IL‐1β, IL‐5, IL‐6, IL‐10, IL‐12β, IL‐13, IL‐17 and TNF‐α (see Table 1 for functions).

Following incubations, the reaction mixture was analyzed using a Bio‐Plex 200 Luminex‐based multiplex analysis system (Bio‐Rad). Unknown cytokine concentrations in samples were determined using Bio‐Plex Manager Software and standard curves derived from recombinant cytokine standards—data were expressed as fold change from control (Al Gadban et al., 2012). Data that were below the assay's range of detection were assigned values of 0.001 (Abolins et al., 2017).

### 2.6 Data analysis

All data were corrected to account for interplate variation by correction against the mixed reference sample. All analyses were conducted in R version 3.6.3 (R Core Team, 2020).

To test for correlation between gene expression levels of all genes expressed by spleen and MLN tissue, and the circulating serum cytokine levels, the nonparametric Spearman's correlation coefficient was used. Spearman's rank correlation coefficients and associated significance levels were generated using the *rcorr()* function in the “Hmisc” package (Harrell Jr., 2020). To control for multiple testing, adjusted *p*‐values were obtained following the Benjamini–Hochberg procedure (Benjamini & Hochberg, 1995), using the *p.adjust()* function. Correlations were deemed to be significant when the adjusted *p*‐value <.05.

Following Jackson et al. (2011), genes and proteins were then grouped into “pro‐inflammatory” (IFN‐γ, IL‐12β, TNF‐α, IL‐1β, IL‐6, IL‐17, Irf‐5 and Tbx21) or “anti‐inflammatory” (IL‐5, IL‐10, IL‐13 and Gata3) response types by principal components analysis (PCA) using the *prcomp*() function. Across tissue types and circulating cytokine levels, the dominant first principal component (PC1) explained a high proportion of variance in the data (>73.49%; see Figure S1). Spearman's correlation coefficient was again used to test for correlations between the pro‐ and anti‐inflammatory gene and circulating protein PC scores.

Following Robertson et al. (2016), genes and proteins were then further divided into more specific response types (Table 2). Where appropriate, PCA was again used to group relevant gene expression or circulating cytokine levels. For all response types in all tissues or circulating protein levels, PC1 explained a high proportion of variance (>68.21%; see Figure S2).

**Table 2:** Levels of immune gene expression from spleen or mesenteric lymph node (MLN) tissue and circulating cytokine levels, measured in wild *Mus musculus domesticus*, were subdivided into immune response type. Measures of immune response were combined using the PC1 scores from PCA (PC1 loadings shown in brackets)

Response type	Tissue measured		
	Circulating cytokine	Spleen	MLN
Innate	IL‐1β	IL‐1β (0.731)	IL‐1β (0.994)
		Irf5 (0.682)	Irf5 (0.109)
Th1	IFN‐γ (0.017)	IFN‐γ (0.222)	IFN‐γ (0.950)
	IL‐12β (0.285)	IL‐12β (0.723)	IL‐12β (0.300)
	TNF‐α (0.958)	TNF‐α (0.604)	TNF‐α (0.086)
		Tbx21 (0.252)	Tbx21 (0.013)
Th2	IL‐5 (0.007)	IL‐5 (0.337)	IL‐13 (0.989)
	IL‐13 (0.999)	IL‐13 (0.481)	Gata3 (0.151)
		Gata3 (0.810)	
Th17	IL‐6 (0.088)	IL‐6 (0.527)	IL‐6 (0.699)
	IL‐17 (0.996)	IL‐17 (0.850)	IL‐17 (0.715)
Treg	IL‐10	IL‐10	IL‐10

## 3 RESULTS

### 3.1 Sample sizes

The total sample size (i.e., number of individual mice) available varied across the genes and circulating cytokines investigated due to missing samples and assay failures (Table 3; Figure S3).

**Table 3:** The sample size (*n*) of expression levels of immune genes expressed by spleen or mesenteric lymph node (MLN) tissue, or the circulating levels of cytokines as measured by multiplex bead assay. Also showing corresponding gene expression level (ΔΔC_T_) and circulating cytokine concentrations (pg/ml), ±Standard Deviation. Samples were obtained from wild *Mus musculus domesticus* on the Isle of May

Target	Source	*n*	Gene expression (ΔΔC_T_)	Cytokine concentration (pg/ml)
Gata3	Spleen	304	3.01 (±14.45)	
	MLN	348	3.24 (±9.64)	
IFN‐γ	Circulating cytokines	323		116.64 (±272.18)
	Spleen	290	1.50 (±3.45)	
	MLN	305	6.87 (±23.21)	
IL‐1β	Circulating cytokines	323		397.09 (±728.81)
	Spleen	303	1.52 (±2.82)	
	MLN	344	1.87 (±6.45)	
IL‐5	Circulating cytokines	323		15.46 (±17.98)
	Spleen	300	2.35 (±6.84)	
IL‐6	Circulating cytokines	321		64.64 (±77.93)
	Spleen	302	2.67 (±8.67)	
	MLN	341	3.46 (±18.42)	
IL‐10	Circulating cytokines	323		138.66 (±143.80)
	Spleen	304	1.94 (±7.09)	
	MLN	343	8.91 (±26.72)	
IL‐12β	Circulating cytokines	322		1,020.17 (±1,106.76)
	Spleen	285	3.13 (±8.31)	
	MLN	241	8.44 (±21.59)	
IL‐13	Circulating cytokines	320		813.44 (±890.47)
	Spleen	291	2.49 (±9.23)	
	MLN	334	9.07 (±29.82)	
IL‐17	Circulating cytokines	323		354.39 (±428.94)
	Spleen	296	3.14 (±13.16)	
	MLN	342	3.78 (±18.77)	
Irf5	Spleen	306	1.72 (±2.66)	
	MLN	325	1.51 (±4.48)	
Tbx21	Spleen	299	1.79 (±3.13)	
	MLN	313	1.73 (±5.04)	
TNF‐α	Circulating cytokines	320		2,895.97 (±3,155.87)
	Spleen	303	2.56 (±6.49)	
	MLN	337	6.33 (±26.33)	

### 3.2 Correlations between all genes and circulating cytokines

**Figure 1:**
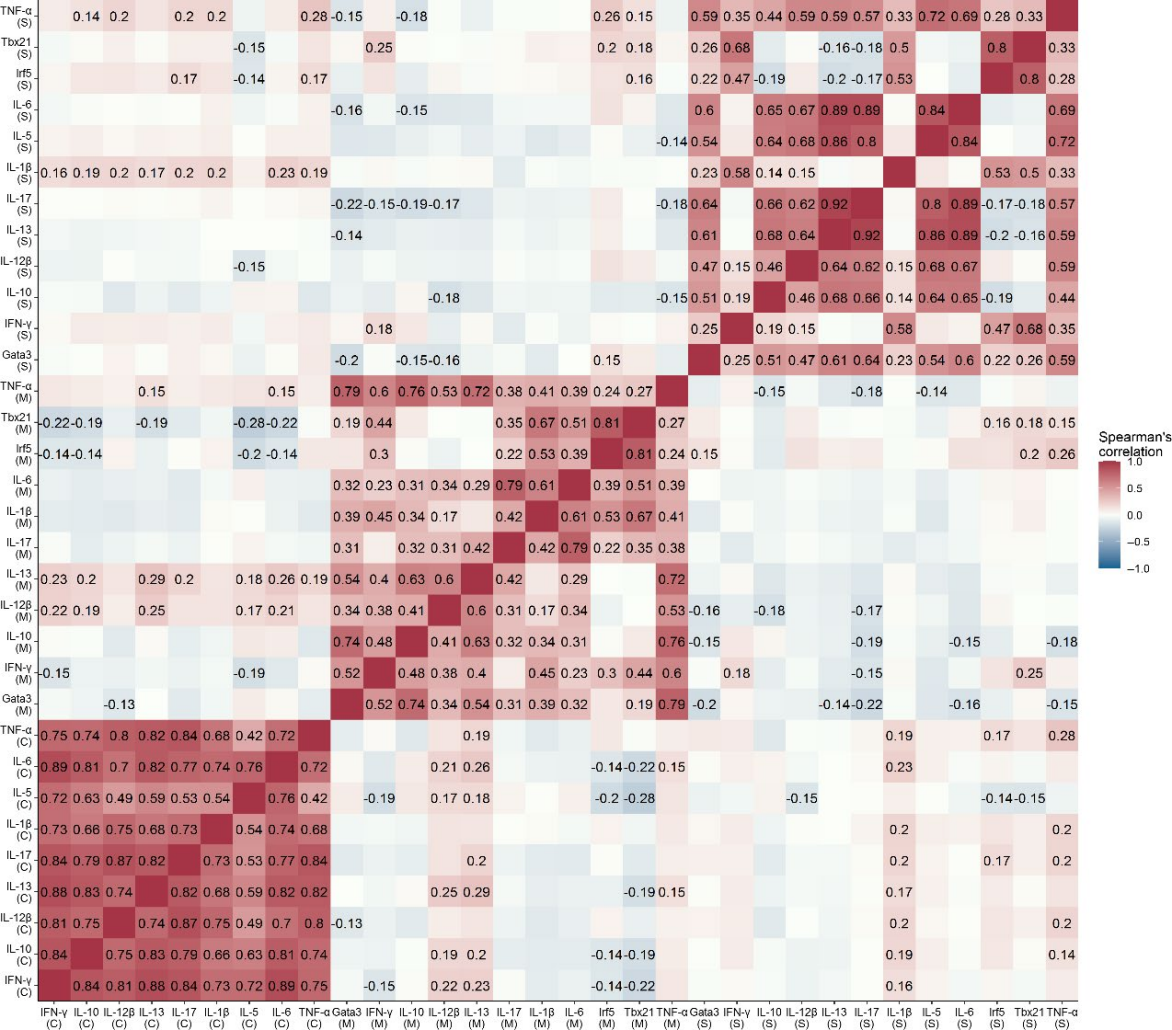
Correlation matrix showing the relationships between immune genes expressed by spleen tissue (S), immune genes expressed by mesenteric lymph node (M) tissue, and circulating cytokine levels measured by multiplex bead assay (C) in wild house mice (*Mus musculus domesticus*) from the Isle of May, Scotland. Labeled in the matrix are the Spearman's correlation coefficients of significant relationships (adjusted *p* < .05)

The strongest correlations came within tissue types for gene expression levels or when comparing the relationship between levels of circulating cytokines (Figure 1). All individual cytokines (36 pairings) were significantly positively correlated. 83.64% (46/55 pairings) of expression levels of genes measured from MLN tissue were significantly positively correlated (adjusted *p* < .023), with 16.36% (9/55 pairings) being nonsignificant (adjusted *p* > .103). Within spleen tissue, 77.23% (51/66) of pairings were significantly correlated (adjusted *p* < .032). These were mostly positive correlations, with the exception of Irf5 with IL‐17 (*r_s_* = −0.171, adjusted *p* > .010), IL‐13 (*r_s_* = −0.195, adjusted *p* =.003) and IL‐10 (*r_s_* = −0.190, adjusted *p* = .003), and Tbx21 with IL‐17 (*r_s_* = −0.177, adjusted *p* =.008) and IL‐13 (*r_s_* = −0.163, adjusted *p* = .016). 22.73% (15/66) of spleen tissue pairings were not significantly correlated (adjusted *p* > .107).

Where significant correlations existed between genes expressed in different tissue types, or between gene expression and circulating cytokine levels, these were weak (*r_s_* ranging from −0.285 to 0.295; see Table S1).

### 3.3 Correlations between inflammation responses

**Figure 2:**
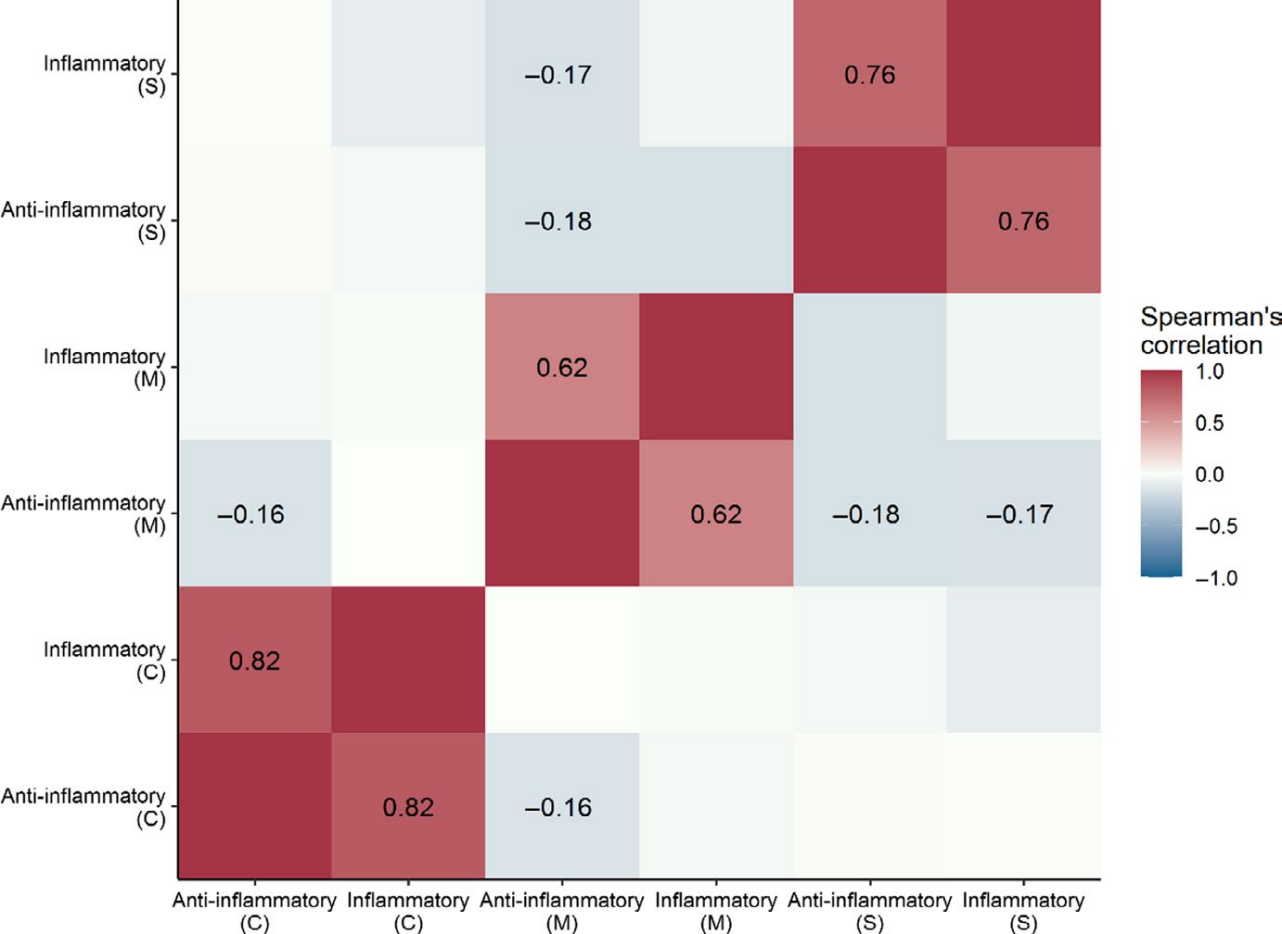
Correlation matrix showing the relationship between the inflammatory and anti‐inflammatory immune response in wild *Mus muculus domesticus* from the Isle of May. Labeled in the matrix are the Spearman's correlation coefficients of significant relationships (adjusted *p* < .05). Immune response was measured using gene expression from spleen (S) or mesenteric lymph node (M) tissue and circulating levels of inflammatory and anti‐inflammatory cytokines measured by multiplex bead assay (C)

When grouped into “inflammatory” or “anti‐inflammatory” genes and proteins, the strongest relationships are again within tissue types (Figure 2). The inflammatory and anti‐inflammatory response types were significantly positively correlated in spleen tissue (*r_s_* = 0.760, adjusted *p* <.001), MLN tissue (*r_s_* = 0.620, adjusted *p* < .001), and circulating cytokines (*r_s_* 0.824, adjusted *p* <.001). In addition to this, the anti‐inflammatory response measured by gene expression from MLN tissue was significantly negatively, although weakly, correlated with circulating anti‐inflammatory protein levels (*r_s_* = −0.159, adjusted *p* = .018), anti‐inflammatory gene expression levels from spleen tissue (*r_s_* = −0.175, adjusted *p* = .018) and inflammatory gene expression levels from spleen tissue (*r_s_* = −0.173, adjusted *p* = .018). No other relationships were significant or strongly correlated (*r_s_* ranging from −0.173 to 0.016, adjusted *p* > .051; Table S2).

### 3.4 Correlations between response type

**Figure 3:**
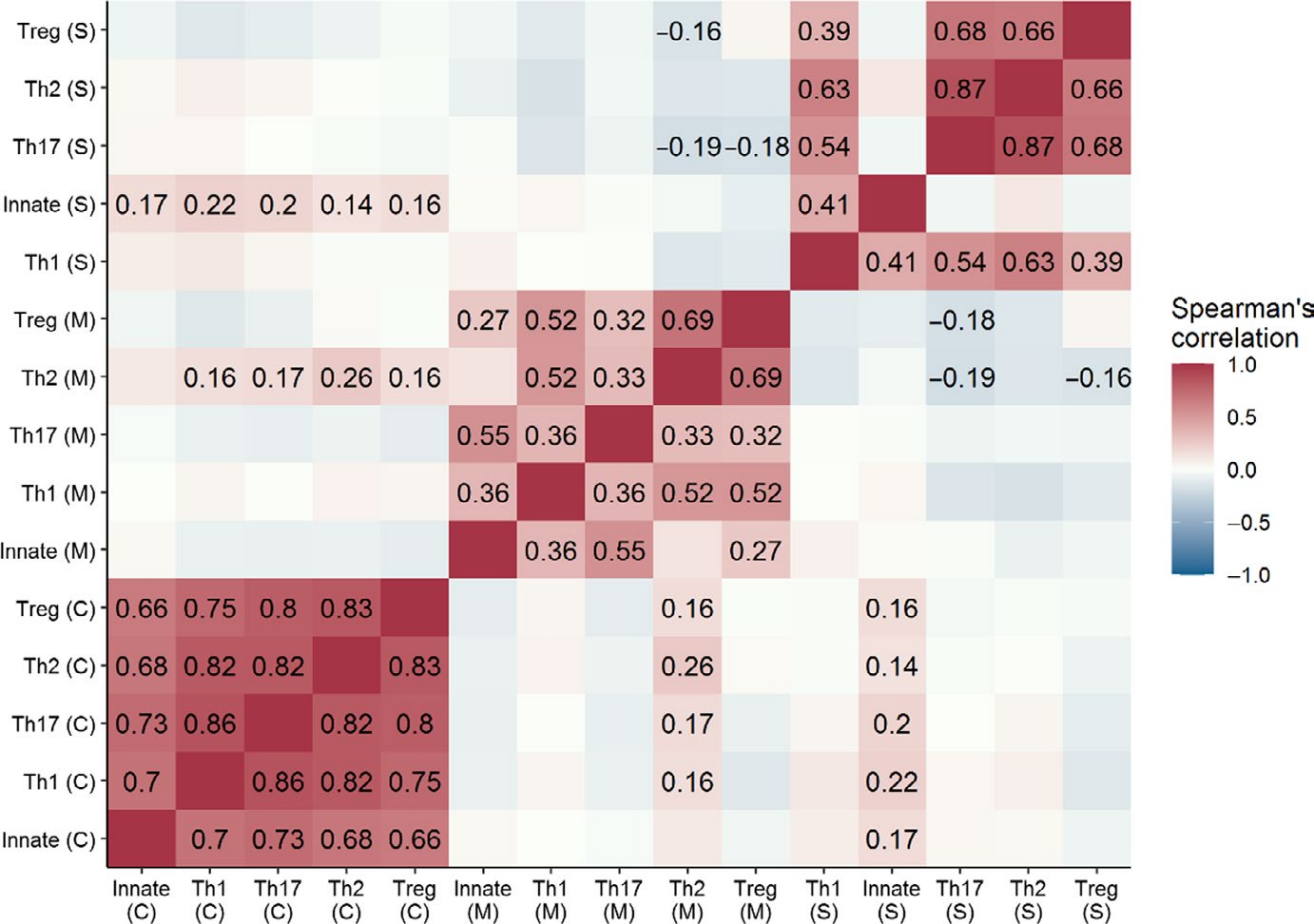
Correlation matrix showing the relationship between immune response type in wild *Mus musculus domesticus* from the Isle of May. Labeled in the matrix are the Spearman's correlation coefficients of significant relationships (adjusted *p* < .05). Immune response was measured by gene expression from spleen (S) or mesenteric lymph node (M) tissue, or by circulating cytokine (C) levels measured by multiplex bead assay

Gene expression and circulating cytokine levels were further split into specific response types (e.g., innate, Th1 etc., see Table 2), following Jackson et al. (2011) and Robertson et al. (2016). The strongest relationships between these functional groupings came within the tissue type from which gene expression was measured, or within circulating cytokine levels (26/38 significant correlations were within tissue type; Figure 3). While there were some significant correlations (adjusted *p* < .049) between different response types as measured by gene expression from different tissue types, or between gene expression and circulating cytokine levels (e.g., between Th17 from spleen and Th2 from MLN), these were often weak relationships (*r_s_* ranging from −0.191 to 0.263; Table S3).

All other correlations were weak and not significant (*r_s_* ranging from −0.168 to 0.132, adjusted *p* > .056). The majority of nonsignificant correlations were between gene expression levels from different tissues, or between circulating cytokine levels and gene expression. However, the innate response measured by gene expression from spleen tissue showed no significant relationship with either a Treg (*r_s_* = −0.056, adjusted *p* = .499), Th17 (*r_s_* = −0.048, adjusted *p* = .598), or Th2‐type response (*r_s_* = 0.107, adjusted *p* = .146), as measured by gene expression from spleen tissue. Further, the relationship between the innate response and Th2‐type response, both measured by gene expression from MLN tissue, was not significant (*r_s_* = 0.132, adjusted *p* = .056) (see Table S3).

## 4 DISCUSSION

Here, we measured the expression levels of key immune genes from two immunologically important tissues (spleen and MLN), and circulating levels of key cytokines in blood, in wild house mice. We found a lack of correlation between the expression levels of immune genes from different tissues, and between gene expression and circulating cytokine levels. Further, when levels of gene expression and circulating cytokines were grouped by response type, the strongest correlations came within tissue types.

Discrepancies between measures of immunophenotype highlight the difficulties of interpreting measures of immune response, particularly in wild animals. While weak correlations are observed in functional groups of cytokines across gene expression and protein levels within a tissue type, there is negligible correlation between genes and their corresponding proteins across tissues. There are a number of potential causes for these discrepancies, both biological and methodological which should be considered when using molecular techniques to assess immune function.

### 4.1 Temporal effects

Cross‐sectional sampling techniques, as were used here, are not ideal when considering delays between transcription and translation, and differences in degradation rates between proteins and RNA. Cell culturing and stimulatory cellular assays can be used to measure the potential immune response to future challenges (Bradley & Jackson, 2008; Robertson et al., 2016), but were not used in this study due to logistical restraints.

### 4.2 Downstream effects

Discrepancies between measures may be indicative of the complexities of the molecular pathways involved, as many of the cytokines analyzed have key roles in inducing or suppressing the expression of different cytokines. A lack of correlation between immune measures in humans was largely explained by post‐transcriptional and post‐translational regulation (Kozak, 2007; Mehra et al., 2003; Nie et al., 2006; Shebl et al., 2010; Shen & Pili, 2008). Immunomodulation by pathogens, such as suppression of Th2 immune function in lab models of *Trichuris* infection (Cliffe & Grencis, 2004), can involve altering host gene expression, rather than the degradation of the cytokines.

The nature of this study means that the immune state is measured across a cross‐sectional sample of a wild population, with the various potential environmental immune stimuli affecting each animal being unknown. While we are here mainly interested in levels of concordance across measures, regardless of the ecoimmunological causes, it should be noted that cytokines are typically only expressed in easily measurable quantities following stimulation, rather than constitutively under typical conditions (Zhu & Kanneganti, 2017). Measurements of these cytokines may be less informative when trying to assess the immune state in a holistic manner, as the level of immune stimulation affecting the host is unknown (Bartoccioni et al., 1994; Nilsson et al., 1995).

Edfors et al. (2016) used an RNA‐to‐protein conversion factor index to increase predictability of protein copy numbers from RNA levels. This, however, is calculated using a whole transcriptome analysis, and so is not appropriate for targeted qPCR studies such as this one. In addition, it is highly unlikely that the weak correlations between gene expression and protein levels seen here would become strong, even with post‐translational or post‐transcriptional correction. Further, this study was investigating the correlation of genes expressed by two different tissue types with secreted proteins, so universally strong correlations were not necessarily expected.

### 4.3 Localization of responses

The gut and the spleen are subject to different immunological challenges. The spleen responds to infections in the blood (Mebius & Kraal, 2005), and can therefore respond to infections throughout the body if immunoactive elements enter the blood and are filtered through the spleen (Bronte & Pittet, 2013). The MLNs drain the gastrointestinal tract and respond to pathogenic or parasitic threats therein. The gastrointestinal tract is a unique immune environment generally, as a complex balance of bacterial communities must be to facilitate normal digestion, while preventing systemic infection or pathology (Houston et al., 2016). Gastrointestinal parasites often reside in the lumen, either wholly or in part, and require unique immune measures in order to deal with infections external to host tissues. Tissue‐specific, localized immune environments may explain concordant differences in immune gene expression.

Laboratory mice experimentally infected with the gastrointestinal nematode *Heligmosomoides polygyrus* showed a decrease in levels of spleen IL‐4 after 21 days of infection, while MLN levels remain elevated up to 70 days after infection (Finney et al., 2007). Increased expression of MHC genes in response to helminth infection might not be detectable in the spleen, but would be detected in tissues local to the infection, that is, intestinal tissues or MLNs (Schwensow et al., 2011).

## 5 CONCLUSIONS

Most studies of wild immunology have focused on non‐model species and so have been restricted to measuring immune gene expression (Jackson et al., 2011, 2014). Protein levels can only be reliably analyzed using sophisticated assays, generally only available for model species such as laboratory mice, *M. musculus*. Although multiplex ELISA assays can be developed for non‐model species, it can be a costly and time‐consuming process. Therefore, gene expression, measured by qPCR, can be a useful tool when it is not possible to measure circulating protein levels. Indeed, while RNA concentrations and protein levels may not correlate, gene expression may provide insight into the types and quantities of immune cell present in a tissue. Regardless, the discrepancy between RNA levels and corresponding proteins should be a consideration that is addressed when reporting results.

Other means of comprehensively assessing immune expression do exist and have potential for applications in wild populations. RNA‐seq provides a whole transcriptome approach, and so any relevant changes in gene expression outside of those target genes can be identified. qPCR, however, has the benefit of being comparatively cheap, thus allowing a higher number of replicates and greater statistical power. Further, the genes of interest selected were based on a priori knowledge of immune function, providing a much more targeted approach.

While the results here show some correlations between different measures of immune response, they highlight that care should be taken when interpreting either gene expression or secreted protein‐level data. Dependent on the precise questions being asked during an experiment, it might be more appropriate to focus on one measure or tissue type than others. For instance, one might be interested in the immune response to a parasite or pathogen localized to a specific tissue, or want to ascertain how immune responses are regulated by upstream gene expression rather than looking at downstream effector proteins. Selecting the measure of immune function should be handled with great care, particularly in wild populations, as factors such as coinfection, seasonal changes, and resource availability can have far‐reaching and overlooked impacts upon immune function. As such, it is advisable to obtain the most comprehensive picture of immunity as is possible in most circumstances.

## CONFLICT OF INTEREST

The authors have no conflicts of interest.

## AUTHOR CONTRIBUTION

**Stuart Young:** Data curation (lead); Formal analysis (lead); Investigation (lead); Methodology (equal); Visualization (lead); Writing‐original draft (lead); Writing‐review & editing (equal). **Jonathan Fenn:** Investigation (equal); Methodology (equal); Writing‐original draft (supporting); Writing‐review & editing (equal). **Elena Arriero:** Investigation (supporting); Methodology (supporting); Validation (equal); Writing‐review & editing (supporting). **Ann E. Lowe:** Data curation (supporting); Investigation (supporting); Methodology (supporting); Writing‐review & editing (supporting). **Benoit Poulin:** Data curation (supporting); Formal analysis (supporting); Investigation (supporting); Methodology (supporting); Validation (supporting); Writing‐review & editing (supporting). **Andrew Maccoll:** Conceptualization (equal); Methodology (supporting); Supervision (supporting); Writing‐review & editing (supporting). **Jan Bradley:** Conceptualization (equal); Formal analysis (supporting); Funding acquisition (lead); Investigation (equal); Methodology (supporting); Project administration (lead); Resources (lead); Supervision (lead); Writing‐original draft (supporting); Writing‐review & editing (supporting).

## Supporting information

Fig S1‐S3

Table S1‐S3

## Data Availability

Data are deposited in the Dryad Digital Repository: https://doi.org/10.5061/dryad.n8pk0p2t8 (Young et al., 2020).
