# Supplementary material for: Relationships between immune gene expression and circulating cytokine levels in wild house mice
Source: Ecol Evol. 2020 Nov 9;10(24):13860–71. doi: 10.1002/ece3.6976 (PMC7771139; doi:10.1002/ece3.6976)
Supplement: Supplementary file 1 — Fig S1‐S3 [file ECE3-10-13860-s001.docx]

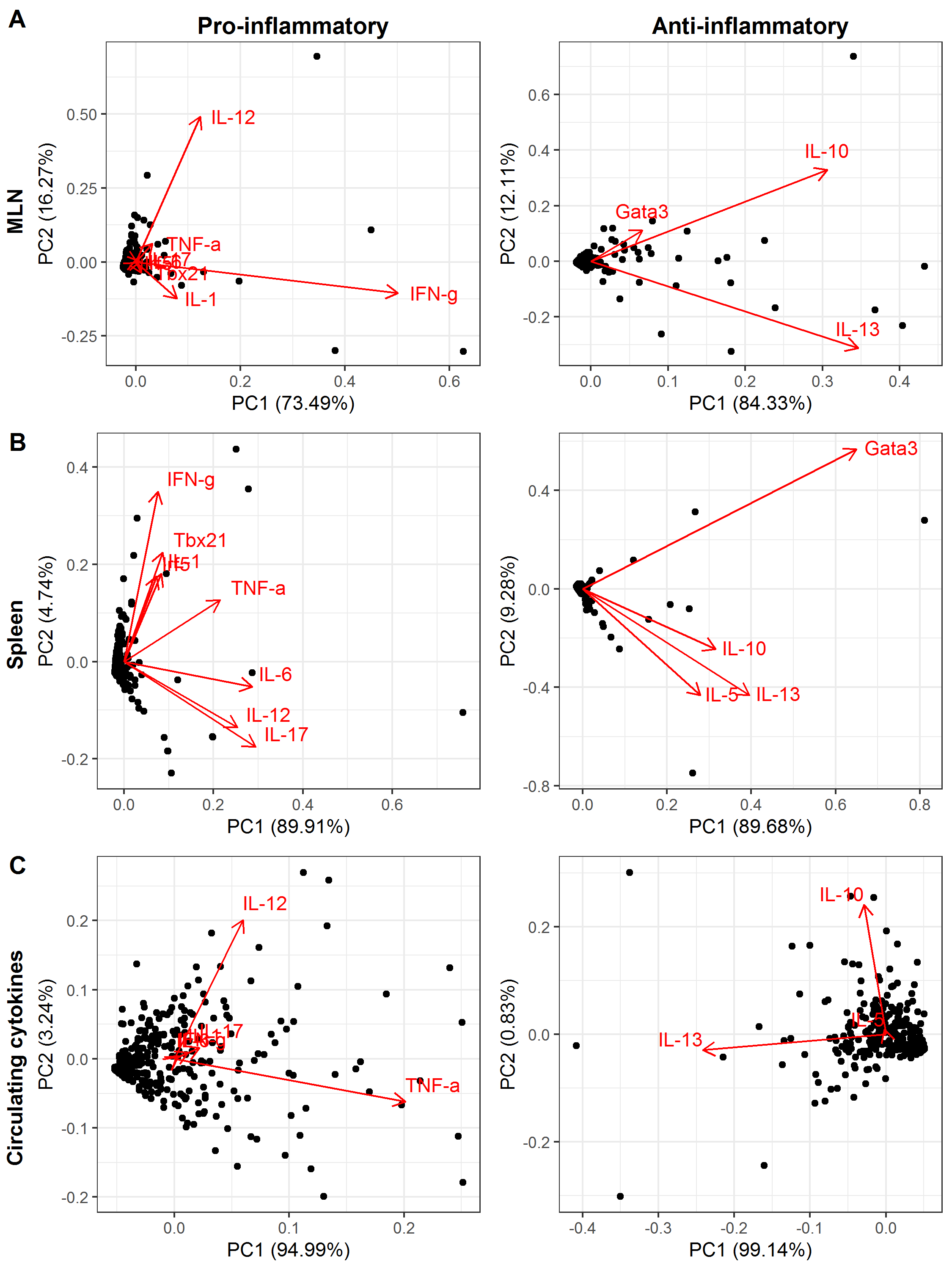


**Figure S1:** PCA biplots of inflammatory and anti-inflammatory immune gene expression (from MLN (A) and spleen (B) tissue), and inflammatory and anti-inflammatory circulating cytokines (C) in wild mice from the Isle of May, Scotland.


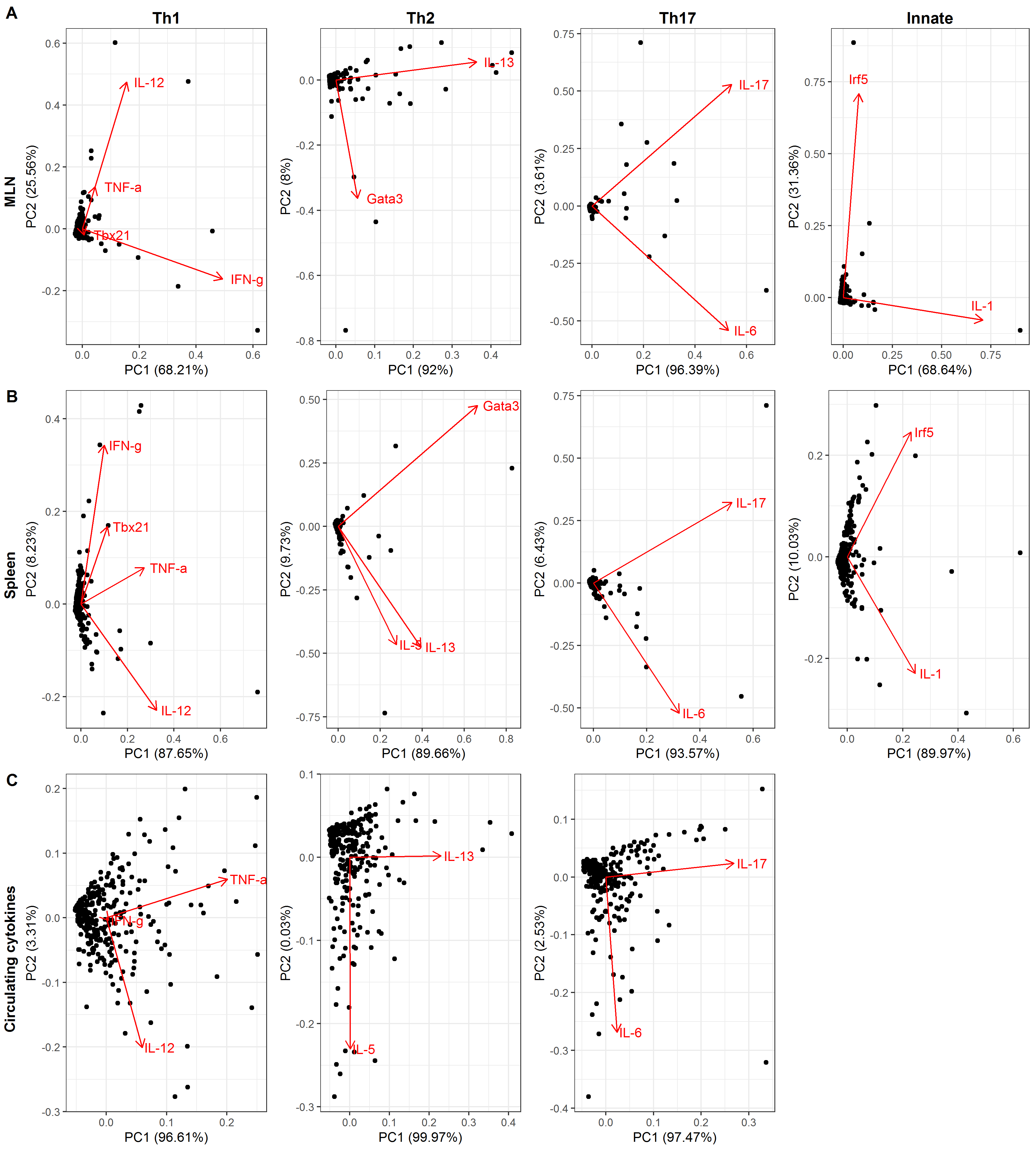
**Figure S2:** PCA biplots of gene expression representing different functional arms of the immune response (from MLN (A) and spleen (B) tissue), and circulating cytokines (C) representing different functional arms of the immune response in wild mice from the Isle of May, Scotland.


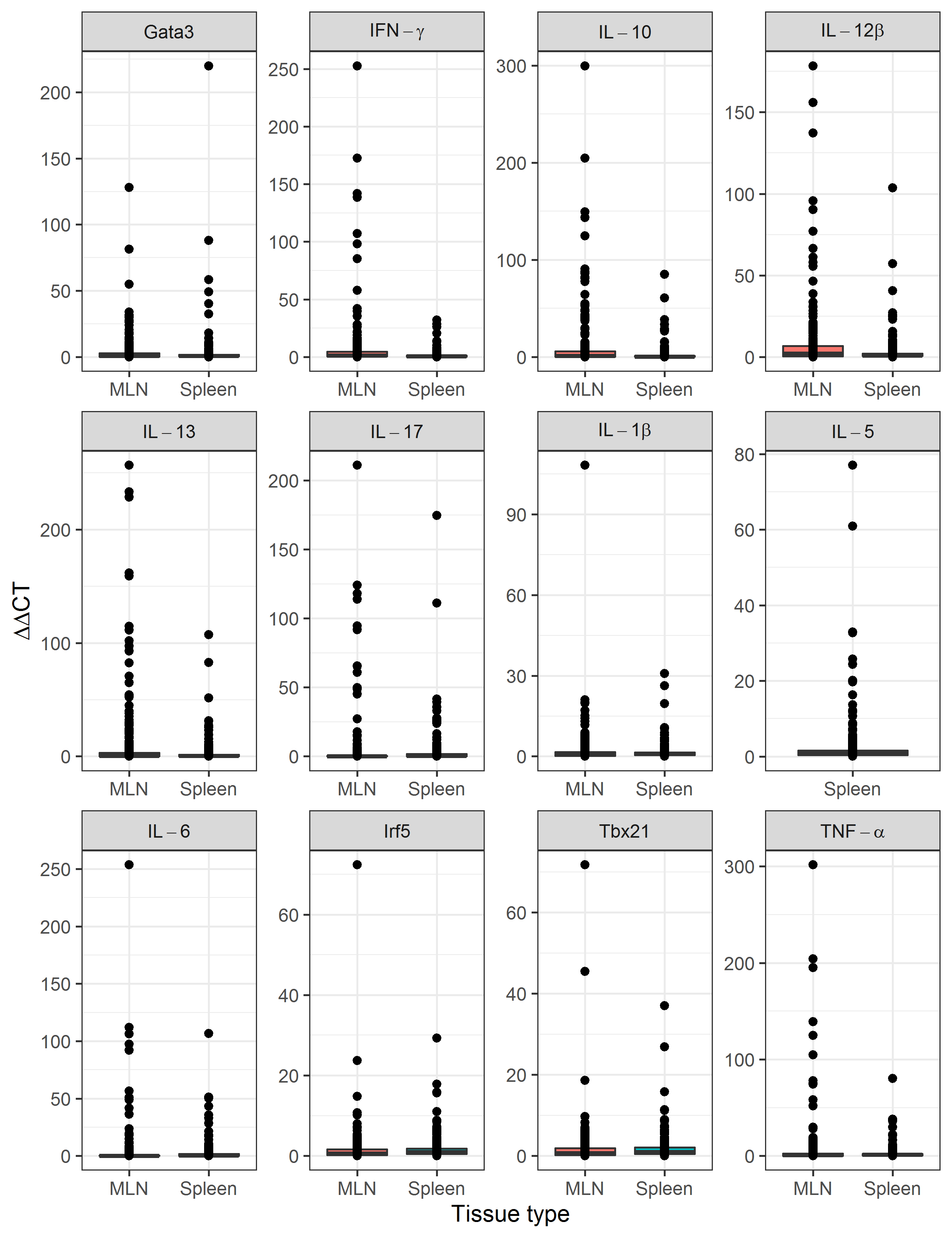


**Figure S3:** Individual variation in immune gene expression (ΔΔC_T_) from spleen and MLN tissue, in wild mice from the Isle of May, Scotland.
